# Supplementary material for: Modeling cryo-EM structures in alternative states with AlphaFold2-based models and density-guided simulations
Source: Commun Chem. 2025 Oct 30;8:317. doi: 10.1038/s42004-025-01751-4 (PMC12575730; doi:10.1038/s42004-025-01751-4)
Supplement: Supplementary file 2 — Supplementary Information [file 42004_2025_1751_MOESM2_ESM.pdf]

Modeling cryo-EM structures in alternative states with AlphaFold2-based models and density-guided simulations

Tatiana Shugaeva[1], Rebecca J. Howard[1,2], Nandan Haloi[1], Erik Lindahl[1,2]

[1]SciLifeLab, Department of Applied Physics, KTH Royal Institute of Technology, Tomtebodävagen 23, Solna, 17165, Stockholm, Sweden.
[2]SciLifeLab, Department of Biochemistry and Biophysics, Stockholm University, Tomtebodavägen 23, Solna, 17165, Stockholm, Sweden.

	Pipeline with k-means clustering, Å	Pipeline with k-medoids clustering, Å
CLR	1.77, 1.8, 1.83, 1.85, 1.99	1.7, 1.77, 1.83, 1.85, 1.86
LAT1	2.02, 2.18, 2.26, 2.34, 2.36	1.66, 1.73, 1.89, 1.92, 1.98
ASCT2	3.96, 4.12, 4.35, 4.42, 4.47	3.28, 3.33, 3.42, 3.64, 3.72

Supplementary Table 1. Cα RMSD to the target for structures closest to each cluster centroid. Values are reported for the cluster that yielded the best-performing structure after running through the whole pipeline. Table with RMSD values for other clusters is deposited on Zenodo (https://doi.org/10.5281/zenodo.14749349)

System	Simulation# (k-means)	RMSD (k-means)	Simulation# (k-medoids)	RMSD (k-medoids)
CLR	5	2.89	5	2.34
	6	2.22	7	**1.46**
	7	1.7	9	2.21
	8	2.18	11	1.66
	9	**1.25**	19	2.5
LAT1	9	6.69	3	**1.64**
	13	4.46	7	1.5
	16	7.65	15	3
	18	2.9	17	2.7
	20	**1.44**	18	3.8
ASCT2	3	8.31	2	**3.46**
	8	**3.65**	9	5.02
	10	6.45	10	6.63
	14	8.6	14	8.96
	17	6.92	20	8.48

Supplementary Table 2. Cα RMSD to the target for the best frames among top-5 simulations ranked by mean cross-correlation. Values corresponding to the simulation with the highest mean cross-correlation are shown in bold.

	% of residues built by ModelAngelo	Cα RMSD before Cryo_fit, Å	Cα RMSD of Cryo_fit result, Å
CLR	85	3.75	3.27
LAT1	43	3.99	2.96
ASCT2	64	13.03	11.73

Supplementary Table 3. Model building results obtained using ModelAngelo and Cryo_fit tools.

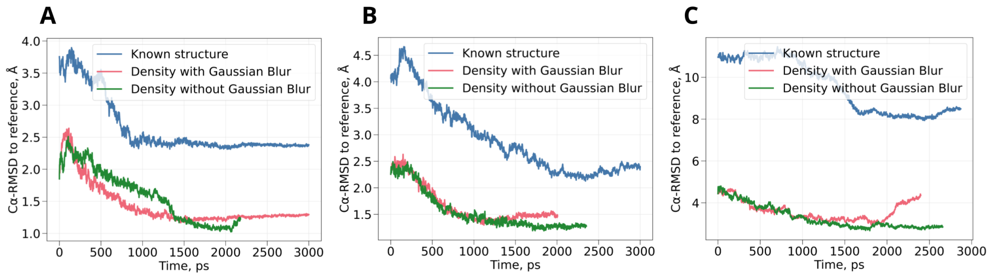

Supplementary Figure 1. Refinement of an alternative protein state by AI model generation in three test systems: (A) CLR, (B) LAT1, (C) ASCT2 with and without Gaussian blur. Plots track Cα RMSD to the target structure during density-guided simulations starting either from the known structure (blue) or best-fit cluster centroid from our generative-AI ensemble with Gaussian blur (red) and without Gaussian blur (green) in CLR.

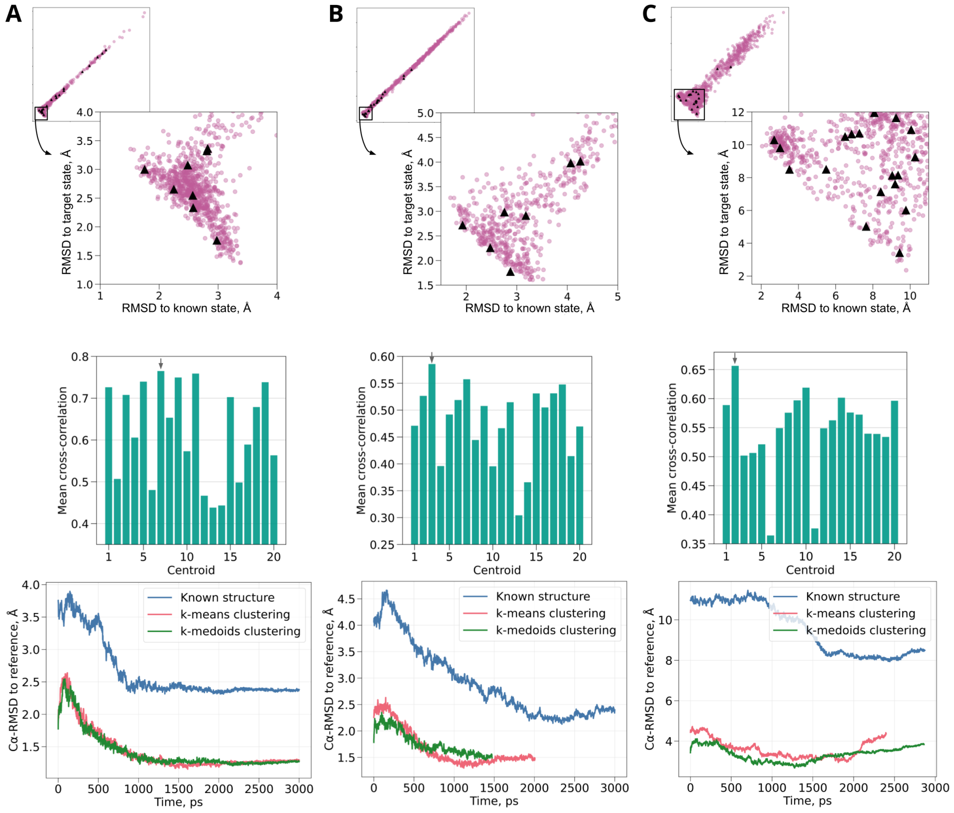

Supplementary Figure 2. Pipeline results with KMedoids clustering of (A) CLR, (B) LAT1, and (C) ASCT2. *Top,* diversity of CLR models from generated ensemble, as indicated by Cα RMSD to target vs. known states. Black triangles highlight the cluster representatives, which were selected using KMedoids clustering algorithm as starting points for density guided simulations. *Center,* mean cross-correlation over density-guided simulations for each cluster representative, with the best-fit representative indicated by a gray arrow. *Bottom,* Cα RMSD to the target structure during density-guided simulations starting either from the known structure (blue), the best-fit cluster representative from our generated ensemble selected by KMeans clustering algorithm (red) or the best-fit cluster representative from our generated ensemble selected by KMedoids clustering algorithm (green).

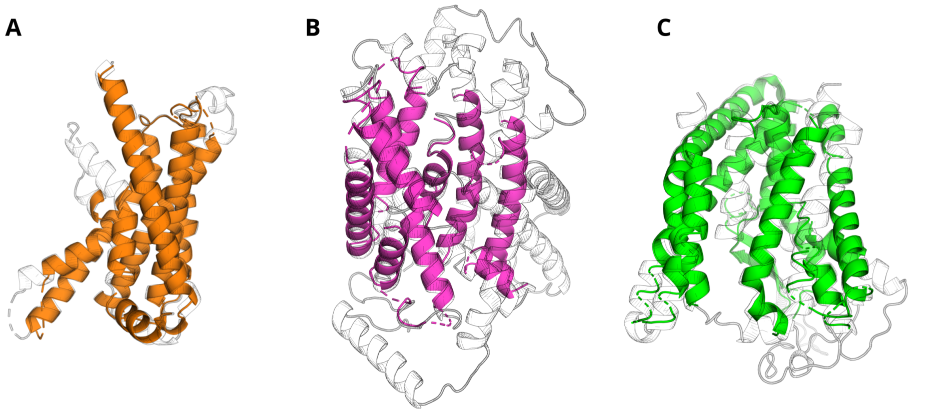

Supplementary Figure 3. ModelAngelo results for three test systems: (A) CLR, (B) LAT1, (C) ASCT2. The target structure is shown as black ribbon outline, while the model built by ModelAngelo is shown with colored ribbons.

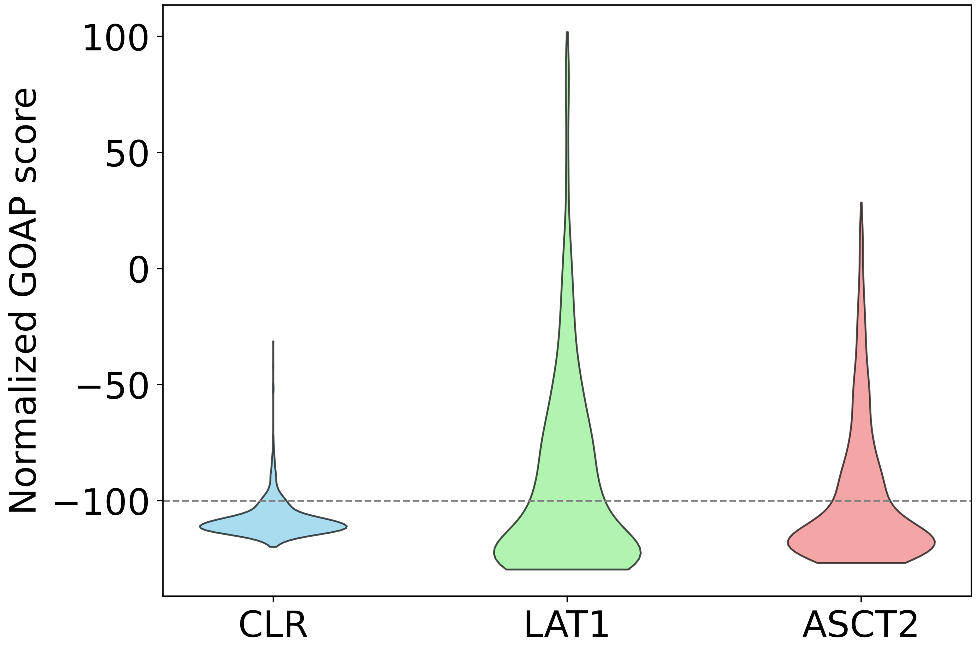

Supplementary Figure 4. Distribution of GOAP scores normalized by protein sequence length, calculated for models produced by AlphaFold2 for each system. The filtering threshold of -100 is depicted as a gray dashed line.

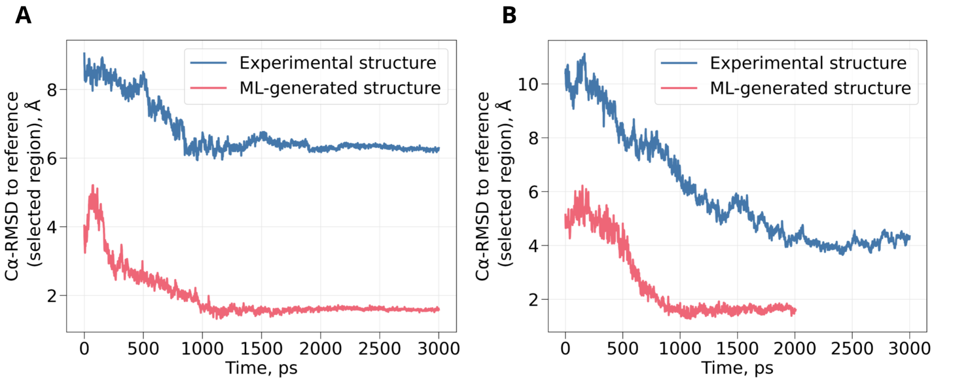

Supplementary Figure 5. Refinement of local conformational changes in two test systems. (A) Cα RMSD to the target structure during density-guided simulations of the known structure (blue) or best-fit cluster centroid from our generative-AI ensemble (red) for the TM6 helix in CLR. (B) RMSD plots as in *A* for the TM1/TM6 region in LAT1.

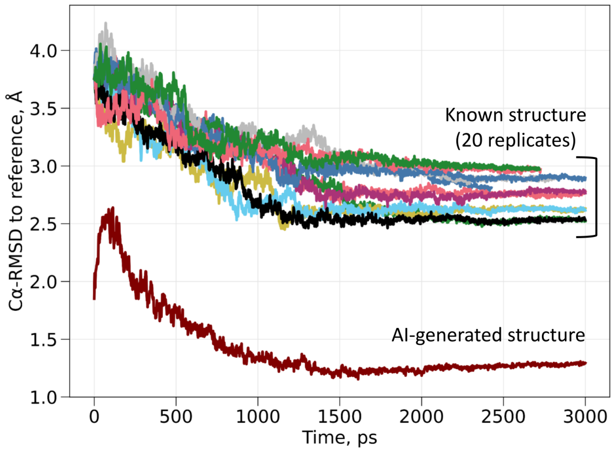

Supplementary Figure 6. Refinement of alternative conformational states in CLR with 20 independent replicates of CLR control simulations. Cα The plot shows RMSD to the target structure during density-guided simulations of the known structure (multicolored) or best-fit cluster centroid from our generative-AI ensemble (maroon) for the CLR test system.

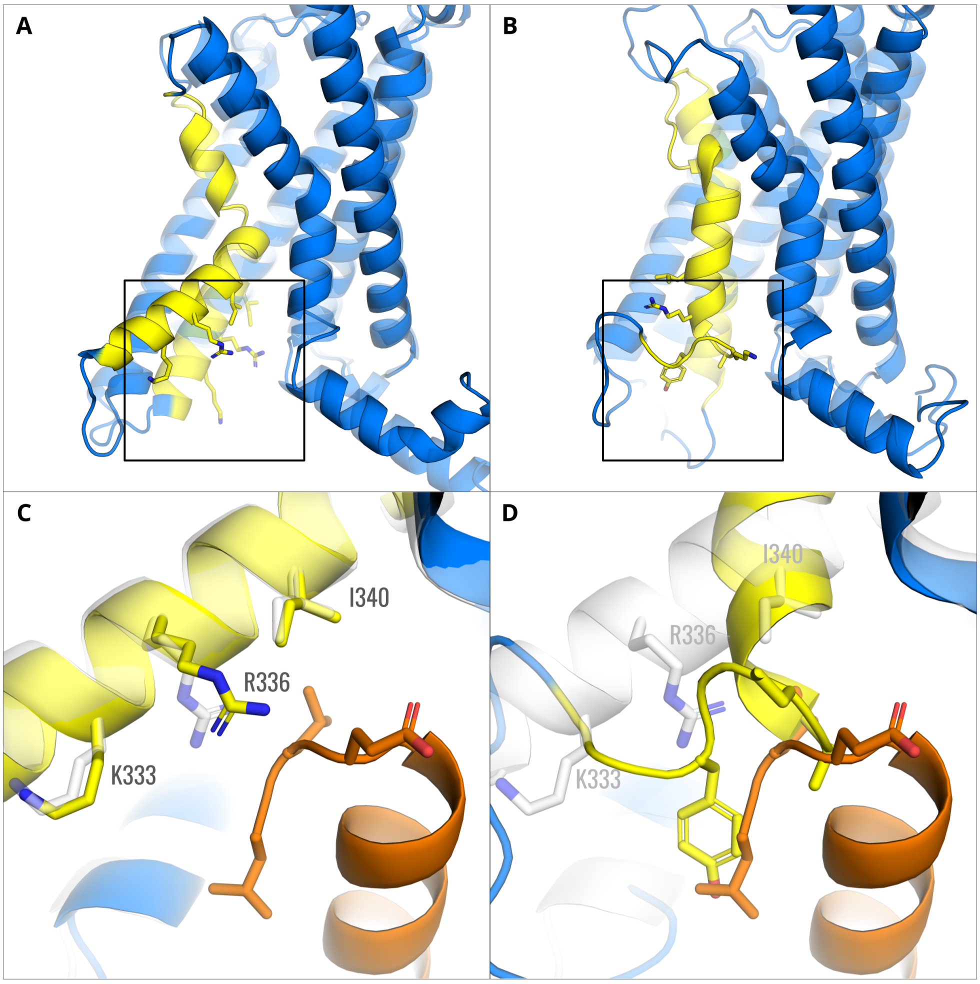

Supplementary Figure 7. Refinement of local conformational change in CLR. (A) Overlay of the final model from our generative-AI approach (opaque) with the corresponding initial cluster centroid (transparent). The bulk of the protein is shown as blue ribbons, with the TM6 region subject to kinking upon G-protein activation as yellow ribbons. Amino-acid residues poised to interact with the Gs-protein α subunit are shown as sticks, colored by heteroatom. (B) Overlay as in *A* of the model from standard known-state fitting (opaque), overlaid with the corresponding inactive structure (transparent). (C) Zoom view of the boxed region in *A*, showing the interface between TM6 and the G protein (orange). The final model from our approach (opaque, colored) is overlaid with the target structure (transparent, white). (D) Zoom view of the boxed region in *B*. The final model from the standard approach (opaque, colored) is overlaid with the target structure (transparent, white).

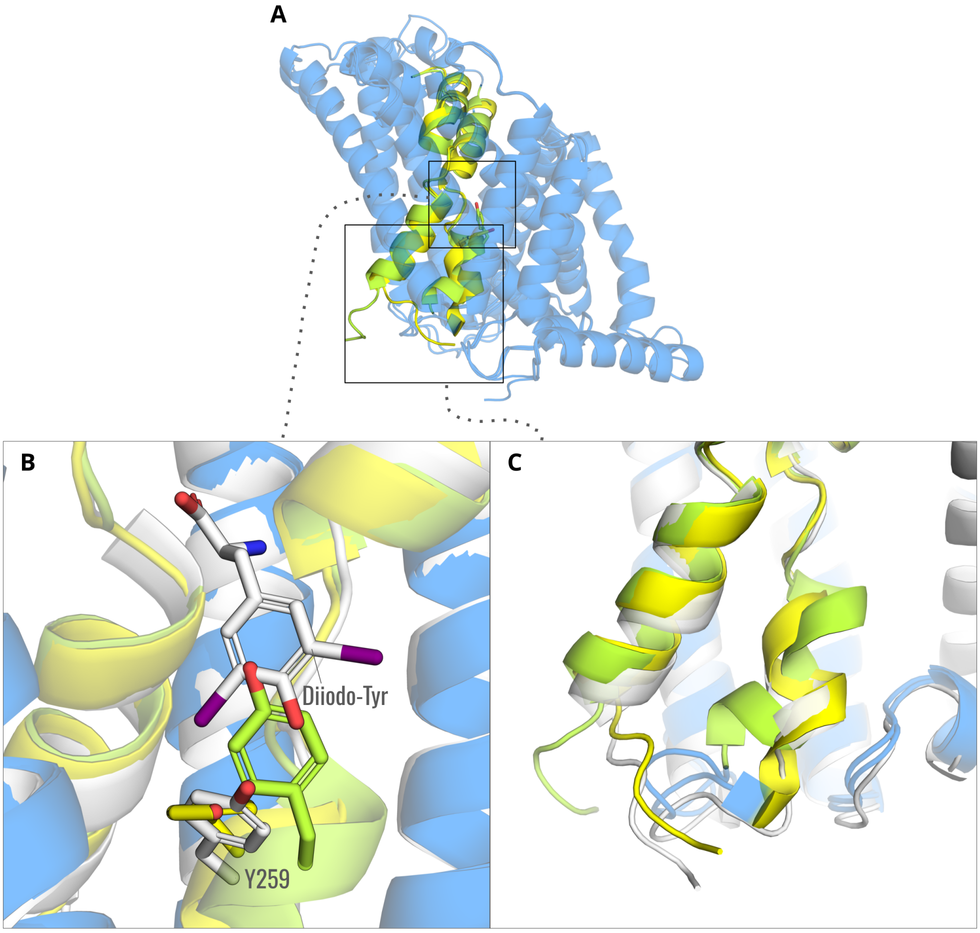

Supplementary Figure 8. Refinement of local conformational change in LAT1. (A) Overlay of final models from generative-AI and standard pipelines. The bulk of both proteins is shown as blue ribbons, with the TM1/TM6 region subject to rearrangement in the inhibited state colored differently for fitting based on the best-fit cluster centroid (yellow) and fitting based on the known structure (green). Residue Y259 is shown as sticks, colored by heteroatom. (B) Zoom view of the upper boxed region in *A*, including the target structure (white ribbons) at the interface between Y259 and diiodo-Tyr (white sticks). Ribbons and sticks for the fitted models are otherwise colored as in *A*, showing prospective clash with the inhibitor in the standard approach. (C) Zoom view of the lower boxed region in *A*, showing the intracellular end of the TM1/TM6 region. Local rearrangements are relatively poorly fit to the target structure (white) by the standard (green) versus generative-AI (yellow) pipelines.

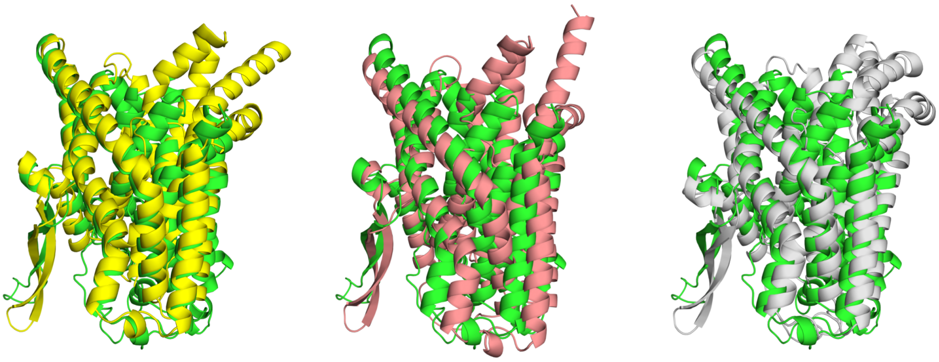

Supplementary Figure 9. Top-3 AlphaFold2 models with highest cross-correlation to the target density. The target ASCT2 structure is shown in green, while AlphaFold2 models are yellow, pink and grey.

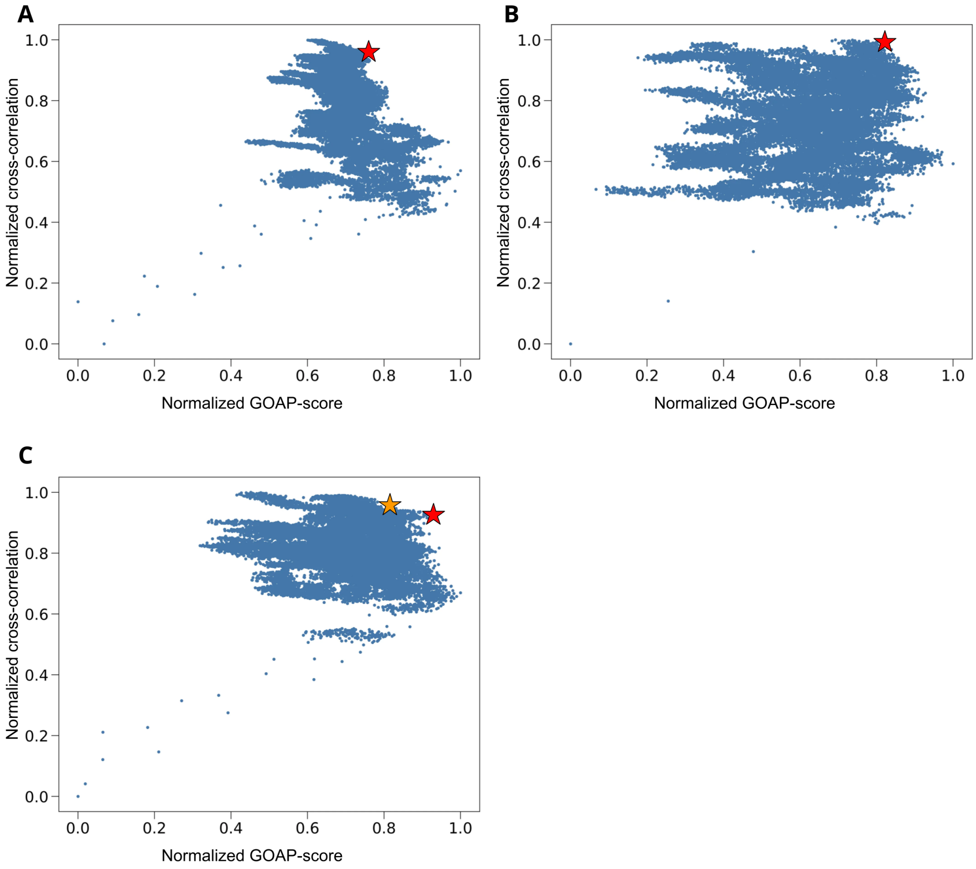

Supplementary Figure 10. Distribution of frames from density-guided MD simulations of (A) CLR, (B) LAT1, and (C) ASCT2. Simulation frames plotted by cross-correlation versus GOAP scores, normalized globally among all simulations of a given system. In each system, the optimal structure based on a compound score calculated using global normalization is marked with a red star; the final structure chosen using normalization within the best-correlated simulation is marked with an orange star. Note orange and red stars overlap for CLR and LAT1.

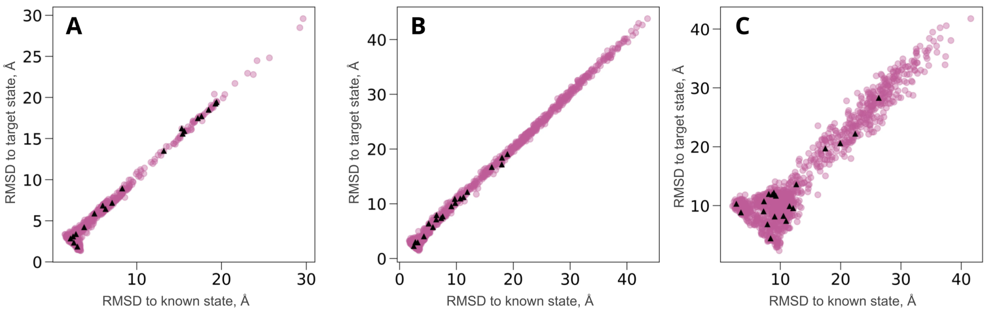

Supplementary Figure 11. Diversity of models from generated ensemble in three test systems: (A) CLR, (B) LAT1, (C) ASCT2. The diversity is indicated by Cα RMSD to target vs. known states. Black triangles highlight the cluster representatives, which were selected as starting points for density guided simulations.
